# Supplementary material for: Assessment of patient perceptions of counselling on oral antineoplastic agents by a dedicated cancer services pharmacist in an outpatient cancer clinic
Source: PLoS One. 2024 Jun 13;19(6):e0304011. doi: 10.1371/journal.pone.0304011 (PMC11175407; doi:10.1371/journal.pone.0304011)
Supplement: Attachment — Submitted filename: REVIEW.docx [file pone.0304011.s001.docx]

**Assessment of patient perceptions of counselling on oral antineoplastic agents by a dedicated Cancer Services Pharmacist in an outpatient cancer clinic**

1. This is an interesting study regarding patient’s perceptions about the CSP. Authors have mentioned that this is the first study in Western Australia. Are there any other studies from other parts of Australia? If so, please kindly reference them. Additionally adding systemic reviews, independent reviews and met analysis will add to comprehensive of your manuscript. Please kindly refer to one such review below. It will be worth to compare and contrast your results from the studies mentioned therein.(A systematic review of the impact of outpatient clinical pharmacy services on medication-related outcomes in patients receiving anticancer therapies)
2. Was permission sought from the authors to use their instrument?

**AIM**: To evaluate patients’ perceptions, experiences and overall satisfaction with dedicated clinical pharmacist consultations in patients treated with oral antineoplastic agents at a major public hospital.

**Background:** Appropriate background and rationale from the literature in the Australian context has been provided for carrying the study.

**Study design**: This study was a retrospective cross-sectional cohort study

**Methods:** These have been described in detail to replicate the study.

The hospital provided a dataset of all patients reported to interact with a CSP between the 1st January 2021 and 28th of February 2022. The dataset included 1528 records and, after removing duplicates, 512 records remained. A randomiser (www.randomizer.org) was used to generate a random sample of 300 patients. Deceased patients (n = 58) were removed and a questionnaire, a patient information sheet and a reply-paid envelope was sent via Australia Post to the remaining patients.

**Participants**: Patients eligibility was mentioned with inclusion and exclusion criteria

Patients, who consulted with the CSP between 1 st January 2021 and 28th of February 2022, were taking at least one OAA and were living, considered eligible for this study. Patients were excluded if they were receiving exclusive IV chemotherapy or sub-cutaneous injections or were under the age of 18 years.

**Sample size: Not applicable**

**Data collection methods, instruments, and measurement:** Validated questionnaire in the English language was used from a previous study.

The questionnaire instrument consisted of four demographic questions regarding age, gender, level of education and English as first language and 16 qualitative questions addressing the benefits of the interaction between the patient and the CSP, self-perceived confidence and knowledge gained, the necessity of the role of the clinical pharmacist and personal adherence and support. These were assessed using a 5-item Likert scale anchored by ‘strongly agree’ and ‘strongly disagree’. The questionnaire was based on a validated questionnaire previously utilized within a similar study before the service was formally established.

**Data management and analysis plan:** Appropriate tests were used in order to assess the study outcomes.

The data were analysed using SPSS software. Categorical data (gender, post code, who referred the patient to the CSP, cancer type, telephone or in person consultation, follow up, intervention, reconciliation) between questionnaire respondents and non-respondents were compared. Chi square and Pearson Correlation tests were used to analyse age and level of education against Likert scale responses. Fisher’s exact test was used for all remaining categorical data due to small sample sizes. For categorical analysis of age, age groups of 70 years of age were used. For analysis purposes the Likert scale responses were grouped as ‘yes’ for strongly agree and agree and ‘no’ for neutral, disagree, strongly disagree and no response. An independent sample t-test was used to compare age between respondents and non-respondents. A p value of < 0.05 was considered statistically significant.

**Ethics**: IRB approval number was mentioned.

**Results:** Tables and results tally with appropriate figures.

**Discussion:** It supports the results with comparison from some countries only. Where there similar studies done in other parts of Australia as the author’s mention that this is first study in Western Australia.?

**Limitations**: Have been mentioned

**References:** More references need to added
